# Aiming Higher: Advancing Public Social Insurance for Long-term Care to Meet the Global Aging Challenge: Comment on "Financing Long-term Care: Lessons From Japan"

**DOI:** 10.15171/ijhpm.2019.121

**Published:** 2019-12-11

**Authors:** Zhanlian Feng, Elena Glinskaya

**Affiliations:** ^1^Aging, Disability, and Long-Term Care Program, RTI International, Waltham, MA, USA.; ^2^Social Protection and Jobs, The World Bank, Washington, DC, USA.

**Keywords:** Long-term Care, Financing, Social Insurance, Universal Coverage, Global Aging

## Abstract

Globally, aging populations are driving the demand for long-term care (LTC) services for a growing number of older people with disabilities or chronic illnesses. A key challenge for policy-makers in all countries is to find a comprehensive solution to financing LTC services to make them widely accessible, affordable, and equitable for all in need. In this commentary, we make a case for LTC policy-makers and reformers across countries to take a long-term vision toward establishing a public, mandatory social insurance model of LTC financing. We first take a hard look at the LTC financing problems and the limitations of existing financing options. We then argue for a public social insurance approach to LTC financing and offer insights into several top-level insurance design features that are key to successful implementation of a public social insurance model, building on the experiences and lessons learned from Japan and other countries that have already "gotten there." We conclude with additional thoughts on the future of public LTC insurance in a global context, including the prospect of spreading this model to middle-income countries.

Globally, aging populations are driving the demand for long-term care (LTC) services for a growing number of older people with disabilities or chronic illnesses. While families continue to shoulder the major responsibility of eldercare in virtually all countries, this traditional source of informal (ie, unpaid) care is increasingly strained and unattainable amidst increasing longevity, rising burden of non-communicable diseases, declining birth rates, shrinking family size, and changing intergenerational relations and living arrangements.^1^ Formal (ie, paid) LTC services, with varying availability across countries, help to fill the gap but typically they are expensive and unaffordable for most older people and their families at prevailing market prices. A key challenge for policy-makers in all countries is to find a comprehensive solution to financing LTC services to make them widely accessible, affordable, and equitable for all in need.

Most countries, even many of the economically developed, rely on a fragmented patchwork of funding sources to operate their LTC programs.^2^ In fact, only a handful of countries have adopted a universal, public social insurance approach to LTC financing. Japan is one of them, along with Germany, Luxembourg, the Netherlands, and South Korea.^3^ In a recent editorial,^4^ Ikegami tells the Japanese story of introducing a German-style, public social insurance LTC financing system in 2000. Mainly from a fiscal perspective, Ikegami offers several useful lessons for other countries interested in establishing similar public LTC insurance programs. For example, reflecting on the difficulties in containing LTC costs—which have resulted from Japan’s comparatively generous LTC benefits inherited from decades of major expansions of social services (eg, health insurance coverage for “social hospitalizations” of older people) before 2000, Ikegami believes that “it would be more efficient and equitable to introduce public LTC insurance at an early stage before benefits have expanded as a result of ad hoc policy.” At the end of the Editorial, Ikegami poses a tantalizing question for policy-makers, including those of middle-income countries: Should public LTC insurance be introduced?

Ikegami hints at a “Yes” to this question and leaves a few cautionary notes. The answer, of course, depends on the country context, and is unlikely to be uniform. In this Commentary, we revisit this important question and make a case for LTC policy-makers and reformers across countries to take a long-term vision toward establishing a public, mandatory social insurance model of LTC financing. Ultimately, we believe this is the most comprehensive and, if properly designed, sustainable solution to the LTC financing problem. However, we are not naïve to suggest that such a model can be introduced and implemented successfully in any country or in any time soon, recognizing each country’s “path dependence” that shapes its policy-making process and policy options.

Below, we first take a hard look at the LTC financing problems and the limitations of existing financing options. We then make the case for a public social insurance model of LTC financing and offer insights into several top-level insurance design features that are key to successful implementation of a public social insurance model, building on the experiences and lessons learned from Japan and other countries that have already “gotten there.” We conclude with additional thoughts on the future of public LTC insurance in a global context, including the prospect of spreading this model to middle-income countries.

## Rethinking the Long-term Care Financing Problem

Broadly speaking, there are two types of financing for LTC services: private and public. Private sources of LTC financing include direct out-of-pocket payments from consumers and third-party payments from private LTC insurance for policy holders. The major problem with private LTC financing is its limited coverage because it is affordable only for people with the means.^5^ For private LTC insurance, particularly, it is important to note that it plays a minimal, or supplemental at best, role even in developed countries with established LTC systems,^3,6^ let alone middle-income countries. In the United States, for example, the private LTC insurance market has imploded to near total collapse over the past 20 years.^7^ Currently, no more than 10% of the adult population in the United States have private LTC policies.^8^ Reasons for the low market demand and dismal take-up rate for private LTC insurance include high prices, Medical underwriting, and individuals’ tendencies to underestimate or deny the future risk of LTC needs.^9,10^ Hence, we take it as a given that private financing is not a viable option for the vast majority of older people in most countries.

This leaves us with public LTC financing for consideration. Public LTC financing exists, more or less and in various forms, in most countries. What differs, and matters the most, is how public funds are organized and allocated and the extent of coverage for people in need of LTC. In grading the strength and desirability of a public LTC financing scheme, one critical consideration is the distinction between universal versus means-tested coverage, which indicates the scope of entitlement to LTC benefits.^3,11^ On one end of the spectrum, only a small number of countries have achieved universal LTC coverage, with LTC services financed primarily through public social insurance (eg, Germany, Japan, Luxembourg, the Netherlands, and South Korea) and/or general taxation (eg, Denmark, Finland, Norway, and Sweden). On the other end are some countries that primarily use means-tested, safety-net approaches to LTC financing (eg, England and the United States). In between are many more countries that have a mixture of these two types of financing methods (eg, Australia, Austria, Canada, France, Greece, Ireland, Italy, New Zealand, Poland, Spain, and Switzerland).

Countries with means-tested LTC financing systems limit publicly covered benefits only to the poor who meet certain eligibility requirements determined by incomes and/or assets. The underlying philosophy is that individuals and families assume the primary responsibility for LTC and that the government should act only as a payer of last resort for people unable to provide for themselves.^6^ In means-tested programs, public funds usually come from taxation and the general government budget, which are often locally based and subject to the vicissitudes of fiscal pressures and budgetary shortfalls. Furthermore, the use of a means-tested, and often stringent, threshold for determining eligibility always creates a group of people who are not poor enough to qualify for public funding and yet not rich enough to pay for the costs of needed care, raising concerns about fairness and equity in LTC access.^3^ The receipt of means-tested benefits could also be accompanied with a sense of stigma.^5^ As such, the limitations of means-tested programs are substantial, leaving large gaps in coverage for the vast majority of middle-class population. These shortcomings are in stark contrast to the advantages of a public, universal-coverage LTC financing model based on social insurance principles, to which we turn next.

## Towards a Public Social Insurance Solution to the LTC Financing Problem

The hallmarks of a public social insurance LTC financing system include broad-based social contributions (typically through employee payroll taxes, often matched by employer contributions and/or supplemented by government subsidies), universal coverage, and mandatory participation.^3^ Underpinning this approach is the philosophy that the government should take the lead in mobilizing resources to ensure that all people with disabilities are eligible for the LTC services they need, regardless of financial status.^6^ The underlying premise is that the financial risks associated with LTC use are so great for the vast majority of older people and their families that a collective arrangement for social protection against these risks is imperative. As such, social solidarity is highly valued, and universal access to LTC is viewed as an entitlement similarly to the entitlement to basic medical care.^2,6^ Since everyone pays into the system and gets benefits from it once meeting certain disability criteria, it essentially creates an entitlement, ensures equitable access, and eliminates the stigma that goes with means-tested LTC support.

It is important to note that a public social insurance approach to LTC financing does not preclude private options. It provides a floor with basic benefits for everyone, and those with the financial means can always have the choice of purchasing private insurance or pay out-of-pocket for desired amenities. We would like to draw a parallel between this model of LTC financing and the “public option” that is commonly applied in healthcare and other areas of social policy. Defined as “a government program that provides some important good or service, like health insurance, at a controlled price,” a public option has two essential elements to serve the dual policy goals of guaranteeing access to important services at a controlled price and coexisting with private provision of the same service.^12^ The first element, referred to as a “baseline public option,” can be structured with an “opt-in default” to achieve mandatory universal coverage with basic benefits available for all, thereby providing some basic measure of equality. The second element, a so-called competitive public option, can be structured with an “opt-out default” to provide voluntary and supplementary coverage by choice.^12^ For example, in the United States, Medicare is a federally administered social health insurance program funded through mandatory contributions from the working-age population, with a standard set of benefits for all people ages 65 or older, which coexists with private Medicare supplement insurance (called Medigap) policies purchased voluntarily by some individuals to help pay healthcare costs that are not covered by Medicare such as copayments, coinsurance, and deductibles. Social Security in the United States is also a federally administered program funded through payroll-based mandatory contributions to provide a basic pension for all retired workers, which coexists with private retirement saving plans like a 401(k) plan.

At a more programmatic level, we suggest several tactical considerations. First, in order for a public social insurance LTC financing program to work and to achieve its intended goals, it has to be mandatory. Voluntary insurance is prone to the well-known problem of adverse selection, whereby older and sicker people in anticipation of needing LTC tend to buy in more often than younger and healthier people do, thereby posing greater risk to the program. Because public social insurance is essentially a pay-as-you-go system and the ratio of the working-age population to retirees is expected to shrink in many countries in the future, mandatory participation is critical for maximizing the risk pool and sustaining the system.

Second, the experiences of Germany, Japan, Korea, Luxembourg, and the Netherlands suggest that the existence of a robust universal public insurance program for medical care would be a great facilitator, if not a prerequisite, for successful subsequent introduction of a parallel LTC financing program. With strategic planning, the existing social health insurance policy framework and infrastructure can be built on to increase the efficiency and lower the administrative costs of implementing the social insurance LTC financing scheme.^13^ We believe this sequencing is particularly important in emerging economies, where a higher-order priority should be given to first meeting people’s medical care needs, and then social aspects of LTC needs. It should also be noted that the financing for medical care is typically separate from that for non-medical LTC services in virtually all countries (perhaps for a good reason, given the distinction between these two types of care, notwithstanding the challenges for integrating them).

Lastly, fiscal conservatives may raise concerns about the costs and administrative burden of a government managed public social insurance program for LTC financing or its long-term sustainability. Several strategies can be employed to mitigate these concerns. For example, cost controls can be achieved through tightening up eligibility criteria, reducing benefits or optimizing the modality of their provision (ie, cash versus in-kind services), pricing regulations, or the combination of them, although many of these measures can be unpopular politically. It would also seem wise to “start low” with the benefit package and increase generosity gradually over time, because rolling back benefits has proven difficult, as seen in Japan. We are also aware of the changing employment structure in many countries—for example, the rise of the gig economy—that makes payroll-based contributions challenging. On balance, however, we believe the societal benefits of a public social insurance LTC financing approach outweigh its potential drawbacks, and with strategic adjustments in program design over time, it can be sustainable in the long run.

## The Prospects of Social LTC Insurance in the Global Aging Context

To meet the aging challenge and rising needs for LTC services, the quest for a comprehensive solution to LTC financing will come up on the policy agenda in many countries, sooner or later. In our view, a public social insurance approach to LTC financing points to the right direction, but the journey to get there can be long. It took decades for Germany and Japan to move away from their means-tested LTC programs to universal public LTC insurance in 1995 and 2000, respectively.^14,15^ There may not be such thing as perfect timing, but the demographic imperative could propel policy actions swiftly. For example, the introduction of Japan’s public social insurance LTC program in 2000 was seen as a surprise, considering its reputation as a weak welfare state, its long tradition of family care, and its decades-long economic stagnation.^15^

There is reason for hope and optimism for public LTC insurance to make inroads in other countries, even in some seemingly hopeless places. In the United States, for example, the political environment generally is hostile to the very idea of public social insurance programs, so the chances for adopting such an approach to LTC financing remain slim at the federal level. But there can be exceptions and innovations at the local level. In May 2019, the state of Washington passed the Long-Term Care Trust Act, which is an old-fashioned public social insurance scheme for pooling funds from workers statewide to pay for their future LTC needs—the first of its kind in the country.^16^ According to the Act, starting in 2022, employees in Washington will pay a mandatory payroll tax of 58 cents per month for every $100 income, which amounts to about $18 a month for the average wage earner. It remains to be seen whether other states will follow suits.

Encouragingly, we also note the diffusion of concepts around public LTC insurance in some middle-income countries. China is a notable example. There, our recent work identified the lack of a systematic financing mechanism as major impediment to the development of LTC services.^17^ In response, in 2016 China launched LTC insurance demonstrations in 15 cities across the country.^18^ All of them are financed by existing social health insurance programs, by earmarking a certain percentage or fixed amount per person from the existing risk pooled funds for LTC services. These pilots signal China’s potential move toward public social insurance as the core LTC financing strategy, following the same strategy adopted for financing healthcare. Given the lack of evaluation, the impact of these ongoing pilots is unclear. If successful, China’s experiment with public LTC insurance can provide useful lessons for other middle-income countries.

## Ethical issues

Not applicable.

## Competing interests

Authors declare that they have no competing interests.

## Authors’ contributions

ZF and EG jointly conceptualized this Commentary. ZF wrote the initial draft. EG provided critical review and revision of the manuscript.

## Authors’ affiliations

^1^Aging, Disability, and Long-Term Care Program, RTI International, Waltham, MA, USA. ^2^Social Protection and Jobs, The World Bank, Washington, DC, USA.
